# Device Modelling and Optimization of Nanomaterial-Based Planar Heterojunction Solar Cell (by Varying the Device Dimensions and Material Parameters)

**DOI:** 10.3390/nano12173031

**Published:** 2022-08-31

**Authors:** Vijai Meyyappan Moorthy, Viranjay M. Srivastava

**Affiliations:** Department of Electronic Engineering, Howard College, University of KwaZulu-Natal, Durban 4041, South Africa

**Keywords:** planar heterojunction, metal-organic frameworks, organic-solar cell, microelectronics

## Abstract

The objective of this work is to model a multi-disciplinary (multi-physics) organic photovoltaic (OPV) using mathematical modeling and analyzing the behavior of a standard planar heterojunction (PHJ) or bi-layer thin-film photovoltaic device, supporting the optimization of an efficient device for future production and assisting in evaluating and choosing the materials required for the efficient device. In order to increase photodiode performance, the device structure and geometrical properties have also been optimized and evaluated. In this work, the effects of varying the device size and transport parameters on the performance parameters of a PHJ OPV structure comprised of Indium Tin Oxide as the anode (ITO), semiconducting single-wall carbon nanotube (s-SWCNT) as the donor, fullerene C_70_ as the acceptor, and Aluminium (Al) as the cathode have been analyzed. The conclusion suggests that a highly effective ITO/s-SWCNT/C_70_/Al PHJ solar cell may be fabricated if the suggested device is appropriately built with a thin layer and a high exciton diffusion length, bi-molecular recombination coefficient, and improved mobility charge carriers, in particular hole mobility in the cell’s donor layer. In addition, the displayed current–voltage (I–V) characteristics of the proposed PHJ device are clearly indicated, with the ITO/s-SWCNT/C_70_/Al combination having the greatest short-circuit current density (*J_sc_*) value of 5.61 mA/cm^2^, open-circuit voltage (*V_oc_*) of 0.7 V, fill factor (*FF*) of 79% and efficiency (*ɳ*) of 3.1%. Results show that the electrical performance of organic solar cells is sensitive to the thickness of the photoactive substance. These results open the path for developing inexpensive and highly efficient solar cells.

## 1. Introduction

Recent interest has focused on organic photovoltaic (OPV) technology because it promises efficient solar energy conversion and a wide range of new, portable applications. However, major performance and device longevity advancements are required for this technology to be successfully commercialized. Protecting the environment from the wastage of materials required for electrical power necessitates the development and construction of new power generation and storage devices, from batteries to power plants. Directly turning sunlight into energy, solar cells can fulfill part of these power demands. Edmond Becquerel discovered the photovoltaic (PV) phenomenon in 1839. It remained a scientific phenomenon with limited device applications for a long period. Silicon PV diodes were accessible after silicon was introduced as the primary semiconductor material in the late 1950s. The refinement of quantum physics, comprehension of the significance of single-crystal semiconductors, and elucidation of p/n junction behavior benefitted solar cell research and innovation. In 1954, Chapin et al. [1] demonstrated a 6% efficient single-crystal Silicon photovoltaics.

Photovoltaics is a frequently utilized renewable energy source for generating power from sunshine. Silicon solar cells accounted for nearly 90% of the worldwide market of various types of renewable energy sources. Mondal et al. [2] later utilized the Cambridge serial total energy package, studying the electrical and optical characteristics of the discovered compound In3–xSe4. Their research has explored the effect of various physical parameters on photovoltaic performance to maximize the cells’ efficiency. The solar cell’s optimal power conversion efficiency (PCE) was 22.63%, *J_sc_* = 38.53 mA/cm^2^, *V_oc_* = 0.703 V, and fill factor (*FF*) = 83.48%. These extensive theoretical estimates indicate that the 2D complex In3–xSe4 may be used in the near future to harvest solar energy. Although silicon solar cells are widely utilized in research, numerous other solar cells are also accessible on the market. To meet the ever-increasing demand for electricity, renewable energy sources are gradually replacing the world’s limited primary energy sources (oil, coal, and uranium). The need for cheap, scalable, carbon-free energy sources has prompted a search for innovative methods of converting solar energy into electricity. Silicon solar cells have more than 90% of the global market share despite their indirect band gap, low light absorption, and high manufacturing temperature of 1400 °C [3].

Later, these novel cell types have broadened the range of potential applications and provided additional routes to lower-cost solar electric production. Hydrogenated amorphous Silicon, Cadmium Telluride, Copper Indium Gallium Selenide and Copper Tin Zinc Sulfide thin-film cells, plasmonic solar cells, and organic cells are among the alternative cells. Organic solar cells (OSC) have various benefits over inorganic solar cells, including low-temperature manufacture, flexibility, and low cost. Recently, researchers’ attention has been drawn to Silicon/organic hybrid solar cells due to their potential to combine the benefits of both organic materials (ease of processing) and inorganic materials (greater charge carrier mobilities and lower exciton binding energies). The organic photovoltaics are predicted to become a substantial source of energy generation due to their benefits over Silicon-based photovoltaic modules, such as flexibility, affordable manufacturing cost, and large-area production methods employing existing industrial roll-to-roll procedures such as coating and printing [4,5]. Extensive research has been performed to boost the efficiency and stability of OPV in order to compete in the solar cell market. Various cell structures and material compositions have been studied, with high PCE demonstrated [6].

The OSCs are employed as a power source in a variety of applications, including flexible sensors and indoor energy storage systems [7,8]. Tang [9] was the first to report thin-film based OSC in the early 1980s, which was composed of perylene tetracarboxylic derivative and copper phthalocyanine. A PCE of about 1% and FF of 0.65 was achieved. Guo et al. [10] developed a single-layer universal OSC model in 2008, and they explored the impacts of charge transport, energy gap, and excitation size on efficacy. Li et al. [11] investigated the effect of cathode work function, exciton generation rate, and temperature, in addition to carriers and field distribution in the organic layer, on the *V_oc_* of a single-layer organic solar cell with a Schottky contact using a computational method. Until the cathode work function approaches the lowest occupied molecular orbit (LOMO) level of the organic material, a decrease in the cathode work function results in an increase in the *V_oc_*. Later, in 2010, Ma et al. [12] used a computer model to examine the impact of the energy barrier between the cathode and the LOMO of the acceptor layer on the carrier density, electric fields, and electric potentials of organic PHJ photovoltaics. The *V_oc_* organic PHJ photovoltaics will be studied further, and our results provide a theoretical foundation for further work.

Lacica and Inganas [13] created a theoretical model to analyze the influence of materials and device factors on mixed heterojunction organic solar cell performance. When the model was applied to devices with a layer of alternating fluorene and fullerene copolymers, it suggested that the restricted hole mobility in the mix is the limiting feature. Donor–acceptor separation suppresses electron–hole recombination relative to Langevin recombination.

Bi-layer photovoltaic current–voltage curves were predicted using a computational model established by Barker et al. [14]. The effect of space charge on the electric field of the device was modeled, along with photogeneration, injection, drift, diffusion, and recombination of charges. They have developed a mathematical model for the dissociation rate as a field function. The rivalry between polaron pair dissociation and recombination was explored to evaluate the short-circuit quantum efficiency. The logarithmic connection between incident power and *V_oc_* has been seen experimentally. This additional intensity-dependent voltage is produced by the field required to generate a drift current, which neutralizes the current due to carrier diffusion away from the interface. Modeling the current–voltage characteristics of polymers, fullerene BHJ solar cells were subsequently accomplished by Koster et al. [15]. Temperature- and field-dependent charge generation, as well as bimolecular recombination, are all explored. The electric field in solar cells based on poly [2-methoxy-5-(3′,7′-dimethyl octyl oxy)-p-phenylene vinylene] -(OC1C10PPV-) and [6,6]-phenyl C61-butyric acid methyl ester-(PCBM-) (1:4 percent) is rather constant since space-charge factors have relatively little effect. Bimolecular recombination under short-circuit conditions results in a loss of only 7% of free carriers. According to the simulation results, PPV/PCBM solar cell efficiency may be increased to 5.5% by increasing hole mobility and decreasing acceptor strength by 0.5 eV. Equivalent circuit models, experimental work, and semi-analytical methodologies have all been used to create several models of PHJ OSC. Cupric Oxide (CuO), an inexpensive material, was studied by Huang and Tang [16] using numerical simulations as a hole transport medium for PHJ perovskite solar cells. When applied to the proposed device architecture, CuO-based HTL shows high photovoltaic efficiency. The absorber’s thickness and optical band gap are also optimized for maximum solar conversion. The structure’s cell *ɳ* is 25.24% at 1.1 eV *V_oc_*, *J_sc_* of 26.32 mA/cm^2^, and FF of 87.14% under optimal operating conditions.

Bendenia et al. [17] analyzed the BHJ inverted OSCs using the effective medium concept. This model is helpful for comprehending the processes involved in charge transport. How the polymer substance of the active layer and the anodic interfacial film HTL influenced the devices’ electrical and optical performance was determined. The PCE of the PTB7 solar cell was 5.73%, which was higher than the 4.88% attained by the P3HT solar cell in the simulation. The inclusion of MoO_3_ to the anodic thin layer PEDOT: PSS also boosts the device’s PCE by 5.92%. The theoretical results provided in the work agreed with the simulated outcomes.

The strong tendency of OSCs to convert photonic light into electrical energy was further investigated by Mishra and Shukla [18] using computational research. The PTB7: PC_70_BM donor polymer was used to build the device. They have evaluated the donor blends temperature- and thickness-dependent PCE. That study contributes to the deliberate intent of the development of organic material architectures for high-performance solar cells. They achieved a PCE of 5.28%. Zhuang et al. [19] later studied the PHJ solar cells with fullerene C_70_ as the electron donor and 1,4,5,8,9,11-Hexa Azatri Phenylene Hexa Carbo Nitrile (HAT-CN) as the electron acceptor in a PHJ structure. They found evidence of efficient exciton dissociation at the C_70_/HAT-CN interface because of the charge transfer from C_70_ to HAT-CN. They acquired a positive result with *V_oc_* = 0.72 V, *FF* = 0.74, and *J_sc_* = 5.3 mA/cm^2^ because the energy levels of C_70_ were well-aligned, and it absorbed a lot of light in the visible range. The ability of fullerenes to move both electrons and holes was proven, and an ohmic contact between MoO_3_ and a fullerene was shown to move electrons.

Most research has been performed on systems that use P3HT as the donor and PCBM as the acceptor. The PCEs for these systems range from 3 to 6% in BHJ and from 0.1 to 3.1% in PHJ, mostly because the contact area between the donor and acceptor is lost [20,21,22]. The BHJ efficiency values listed above usually refer to rigid substrates such as glass. However, when the devices are built on plastic or flexible substrates, the efficiency drops dramatically (0.08–2.25%) because the electrode on the substrate has a higher surface resistivity and plastic is less transparent to light than glass [23,24]. Plastic PHJ solar cells have not advanced significantly in recent years. Despite the obvious processing and application benefits, widespread adoption of these devices has been slow. To be sure, large-area plastic solar cells are easier to produce when PHJ solar cells are used instead of BHJ solar cells. Because BHJs’ morphology is controlled by out-of-equilibrium phases, often via annealing processes, this might be challenging. Plastic substrates may be used for a wide variety of purposes, including artificial retinas, solar sails, and anything else where lightweight design and seamless tissue integration are desirable [25,26]. The development of plastic PHJ devices for practical use is hindered by the problems of donor–acceptor interface loss and employment of plastic substrate; both have been the topic of extensive research. New materials with enhanced air stability, carrier mobility, and considerable absorption across a broad spectrum have been the primary research focus [27,28,29].

Hellgren et al. [30] reported nanoindentation measurements to show that all BxCyNz films exhibited a highly elastic response independent of elemental composition. Broitman et al. [31] compared the uptake of water of amorphous carbon nitride (a-CNx) films, widely used as protective overcoats in computer disk drive systems, with fullerene-like carbon nitride (FL-CNx) and amorphous carbon (a-C) films. The experimental results show that dangling bonds are much less likely in FL-CN*_x_* than in a-CN*_x_* and a-C films.

Jahangir et al. [32] utilized general photovoltaic device model (GPVDM) software, a 3-D photovoltaic device model, used to observe the outcomes of PTB7: PC70BM-based organic solar cell. The electrical simulation via GPVDM has been performed at different active layer thicknesses and charge carrier mobility. The optimum efficiency of OSC is obtained at 200 nm active layer thickness and carrier mobility of 2.46 × 10^6^ m^2^/Vs. Yakimov et al. [33] demonstrated high *V_oc_* OPV cells that incorporated two, three, or five stacked, thin heterojunctions consisting of Cu-phthalocyanine as a donor, and 3,4,9,10 perylenetetracarboxylic bis-benzimidazole as an acceptor using SYNOPSYS solar simulation software. The power conversion efficiencies of the two and three cells under one sun, AM 1.5 illumination values are more than twice that of a comparable single-junction cell based on the same materials.

Mishra et al. [34] utilized Silvaco TCAD simulation software to simulate and analyze the OSC by introducing the stone wall defect in a carbon nanotube (CNT). They utilized Graphene/PEDOT: PSS/Perovskite/PCBM + CNT/Al as the base structure. The simulation results indicates that the difference between the efficiency in the presence of stone wall defect and without the defect in CNT. Mehrabian et al. [35] demonstrated the OPV with poly 3-hexylthiophene (P3HT) absorber layer, designed and simulated using electronic device simulator. The simulation result showed an *V_oc_* of 0.32 V, a *J_sc_* of 0.07 mA/cm^2^ and a *FF* of 29.5%, resulting to a PCE of 0.005%. Petterssona et al. [36] performed electrical modeling of Cu(In,Ga)Se2 solar cells with Zn1xMgxO buffer layers. By contrasting simulation findings with measurement data, several device models are enacted and evaluated. These cells’ low-temperature properties as well as their behavior under ambient light and dark-light cross-over are analyzed. Models using acceptor states at the absorber–buffer interface produce the best agreement with data on ZnO and Zn0.83Mg0.17O cells. To replicate measurement data, a wideband-gap surface defect layer is not required. Khadka et al. [37] demonstrated the optoelectronic properties of perovskite PV devices with and without annealing the layer in a methyl ammonium chloride vapor environment were investigated and quantitatively compared. The obtained results indicated MACl-treated devices are more efficient, with a best efficiency of 15.1% and a modest standard deviation (std.) (0.50%), and have better stability than non-MACl-treated devices, which have a best efficiency of 12.4% and a std. of 0.66%.

In previous research work, Moorthy et al. [38,39,40] analyzed the BHJ and PHJ topologies utilizing various nanomaterials and successfully fabricated and evaluated a subretinal implant NPD device. The nanomaterials used in that work increased the BHJ performance for subretinal implants. In this present research work, the authors continue the previous work [38,39,40,41,42] by proposing an improved PHJ device using different nanomaterials in comparison with the reported works. In this present research work, the PHJ-based OSC structure (ITO/s-SWCNT/C_70_/Al) has been analyzed. The device is then further improved to examine the impact of various factors on output performance. This research focuses on developing efficient OSCs with excellent stability and longevity. In addition, the effect of various parameters such as layer thickness of the device and transport parameters have been realized. This paper has been organized as follows: Section 2 contains the proposed photovoltaic devices’ structure and the methodology implemented. Section 3 describes the modeling of the PHJ cell. Section 4 contains a comparison, optimization, and evaluation of efficient PHJ structures. Finally, Section 5 concludes the work and recommends the future aspects.

## 2. Methodology and Mathematical Modeling with Operation and Device Physical Principles of Planar Heterojunction

An electrical device simulator is a useful tool that predicts the behavior of a proposed PHJ OSC. The methodology uses drift-diffusion based transport equations to determine the current–voltage (I–V) curve. The I–V characteristics serve as the foundation for the solar cell’s performance computation, including the *FF*, *ɳ*, *J_sc_*, and *V_oc_*_,_ among other metrics. This has been performed to discover how device characteristics such as carrier mobilities, work functions, and layer thickness affect these performance metrics, most notably the efficiency. Therefore, the instrument is an ideal resource for creating the most effective OPV cells.

**Figure 1:**
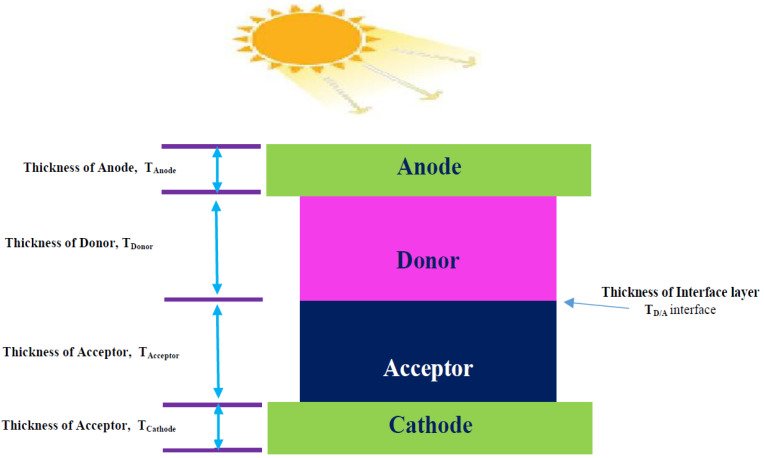
Schematic representation of a PHJ OSC.

A bi-layer morphology (PHJ) is the structure that is mimicked in the tool. The schematic representation of a bi-layer/(PHJ) OSC is depicted in Figure 1. This structure is created by sandwiching the Donor (D) and Acceptor (A) organic semiconductor layers between two electrodes with opposing work functions [43,44].

The process by which an OPV works may be divided into four distinct phases. (i) When light strikes the photoactive layer of an OSC, it is absorbed, a process known as photon absorption. The tool works on the assumption that sun energy with an air mass 1.5 (*AM1.5*) spectrum is incident. Then, using the transfer matrix method [45], they calculated the total quantity of absorption that happened in each cell layer. The complex refractive indexes and the thicknesses of the various layers in the cell contribute to the absorption profile.

When an organic semiconductor absorbs a photon, an exciton is generated. Due to its neutral charge, the exciton may easily diffuse across the active layer. Exciton ion diffusion in the device is represented as:

(1) $$D_{ex}\nabla^{2}n_{ex}=G_{ex}(r)-R_{ex}(n_{ex})$$

where *G_ex_* is the creation of excitement, *R_ex_* is the recombination of exciton_,_ *n_ex_* is the exciton concentration, and *D_ex_* is the coefficient of exciton diffusion.

Equation (2) is used to express the exciton diffusion length *L_ex_* and *τ_ex_* is the lifetime of exciton, which is used to calculate how far an exciton may go without recombining or deteriorating into its component parts.

(2) $$L_{ex}=\sqrt{D_{ex}\times \tau_{ex}}$$

When the exciton is divided down into its constituent parts, the holes pass through the donor, and the electrons pass through the acceptor. The difference in work-function between the two electrodes is what produces the electric field that passes the active layer, which promotes carrier transport.

For the purpose of describing the carrier transport system, the following equations have been used:

Drift-diffusion transport equations [46,47]:

(3) $$J_{e}=e\mu_{e}n(X)E(X)+eD_{e}\nabla n(X)$$

(4) $$J_{h}=e\mu_{p}p(X)E(X)-eD_{p}\nabla p(X)$$

where *µ_p_* and *µ_n_* are the hole and electron mobility, resoectively, *D_p_* and *D_e_* are the hole diffusion and electron diffusion coefficient, respectively, *n(X)* and *p(X)* is the electron and hole Concentration, respectively, and *E(X)* is the electric field. To calculate the electric field indicated by *E(X)*, first solve the Poisson equation represented by Equations (5) and (6) while keeping the transport equations self-consistent.

Boundary conditions:

(5) $$n(d)=n_{Cathode}=N_{c}\exp\left(-\frac{\Phi_{c}-x_{acceptor}}{KT}\right)$$

(6) $$p\left(0\right)=p_{Anode}=N_{v}\exp\left(-\frac{\Phi_{A}-x_{donor}}{KT}\right)$$

where *Φ_C_* and *Φ_A_* are the cathode and anode work function, respectively, *x_acceptor_* and *x_donor_* are the LUMO level of acceptor and donor, respectively, and *N_c_* and *N_v_* are the effective density of states. The recombination is effective at the interface region between donor and acceptor. Let us consider the Equations (5) and (6) that the concentrations of electron and hole at the interface are:

(7) $$n(\frac{d}{2})=n_{I}$$

(8) $$p(\frac{d}{2})=p_{I}$$

It is required to examine the flux balance in order to obtain an equation for the current density represented as:

(9) $$J_{e}=J_{h}=J_{rec}-J_{gen}=q\gamma (n_{I}p_{I}-n_{i}^{2},\mathrm{int})-J_{ex}$$

It is feasible to calculate the power density based on the current density using the formula:

(10) P=V×J

Therefore, to compute *FF*, *ɳ*, *J_sc_*, and *V_oc_*:

Fill factor:

(11) $$FF=\frac{P_{\max}}{J_{sc}\times V_{oc}}$$

Efficiency:

(12) $$\eta =\left|\frac{P_{\max}}{E}\right|\times 100\%$$

Short circuit current density:

(13) $$J_{sc}=qG(L_{n}+L_{p})$$

Open circuit voltage:

(14) $$V_{oc}=\frac{nKT}{q}\ln\left(\frac{I_{L}}{I_{o}}+1\right)$$

By defining the thicknesses of the various layers and other physical features, the aforementioned equations may be utilized to simulate the suggested structures that were produced using the simulation tool. In order to acquire the I–V characteristics, the simulations must first be completed. The suggested PHJ device was subjected to numerical simulations using the *AM1.5* spectra while it was heated to a temperature of 300 °C. The parameters acquired from I–V characteristics are the current–density and voltage characteristics, which have been utilized in this work.

## 3. Device Modeling and Simulation

In this work, the cathode is made of aluminum metal, the electron donor is a semiconducting single-wall carbon nanotube (s-SWCNT), the electron acceptor is C_70_ fullerene, and the anode is made of ITO. Glass or flexible substrates are used in the processing of these materials to increase the device’s efficiency and transparency. The effectiveness and key parameters of the proposed design and its attributes are examined using an electronic device simulator. The active layer materials s-SWCNT and C_70_ are chosen because of the thermal stability. s-SWCNT remains stable at temperatures T from 300 K up to 2000 K. C_70_ fullerite is more stable than C_60,_ the C_70_ remains stable at temperatures T from 300 K up to 1050 K [38,39,40]. Hydrogen bonding in PHJ solar cell plays a major role, this helps to improve the performance of the device. In some cases, improved device performance had been demonstrated when hydrogen bonding was employed in PHJ solar cells. The presence of hydrogen bonding moieties on the electron acceptor fullerene derivatives may provide further insights into the effect of hydrogen bonding on BHJ solar cell devices. As with the electron donor material, the acceptor should ideally phase separately on a suitable length scale to allow maximum ordering and thus, charge can be transported effectively in continuous pathways to the electrodes and the recombination of free charges can be minimized.

**Figure 2:**
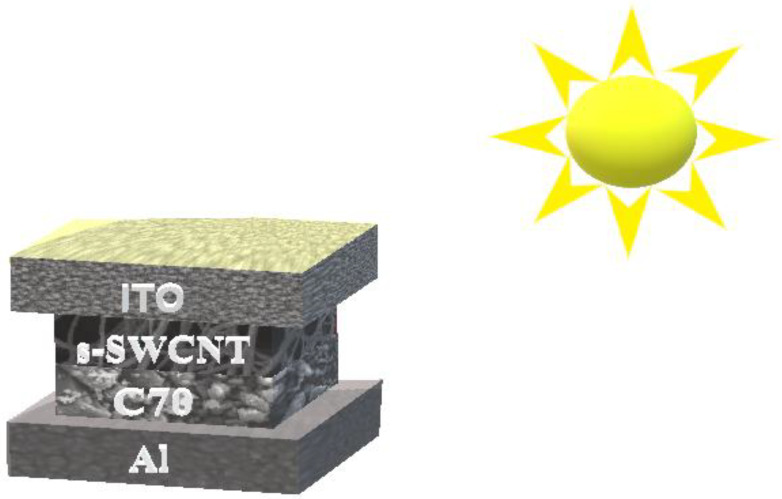
3D schematic view of the proposed and simulated PHJ structure.

Figure 2 depicts the proposed cell structure of the PHJ OSC. There are four layers in total in the electrical model, one of which is the “active” layer. According to Figure 2, the order of these layers is as follows: ITO/s-SWCNT/C_70_/Al. The organic s-SWCNT/C_70_ solar cells are modeled and employ parameters extracted from experimental results. The active layer of a device absorbs photons, separates, and transmits charges to an electrode. Excitons are created by a process that involves the diffusion of free carriers and uses ITO and aluminum electrodes. Materials for the electrodes and the active layers are deposited sequentially on top of one another in this arrangement to form a material junction. Therefore, this type of solar cell is known as a bi-layer junction solar cell.

## 4. Parametric Analysis of the Proposed Model

Nanomaterial-based PHJ organic photodiode cells with a hybrid architecture have been developed in this research work. It enables them to examine and contrast the results of devices with varying geometries and material properties. The suggested device, which incorporates PHJ structures, has been modeled to enhance its performance.

To maximize conversion efficiency, it is necessary to understand the photo physics at work in the cell layout and optimize the device layer accordingly. Optimizing the device is useful for cutting down on material use and increasing performance characteristics.

This modeling’s primary goal is to optimize the layer thicknesses of the device, transport parameter, and energy band parameters to achieve the highest *FF*, *ɳ*, *J_sc_*, and *V_oc_*. Consequently, the proposed device designs depicted in Figure 2 have been designed for simulation tests and the effects of various layer geometries, transport parameters, and energy band parameters on the above-mentioned device performance characteristics. The findings acquired for different structures have been compared in order to determine the optimal device dimensions for the device that provides the best overall performance.

### 4.1. Analysis of the PHJ Solar Cell’s Performance by Varying Layer Thickness

This has been performed for the effect of five different layers, (i) the cathode layer, (ii) acceptor layer, (iii) donor layer, (iv) donor–acceptor interface layer and (v) anode layer. Each of these layers is critical to the device’s overall performance. Therefore, optimization of these layers is vital for developing an efficient photovoltaic device. The active layer (donor–acceptor layer) of the s-SWCNT/C_70_ solar cell contains separate donor (s-SWCNT) layer and acceptor (C_70_) layer. The light I-V characteristics of the s-SWCNT/C_70_ PHJ OSC have been simulated by varying the thicknesses of the anode, cathode, and active layer [48].

To conduct a comparative analysis and improve each PHJ structure’s performance, the authors initially studied the active layer performance by implementing three strategies: acceptor layer, donor layer, and equal proportion of donor–acceptor layer thickness variation. This study has been conducted to optimize the active layer.

**Figure 3:**
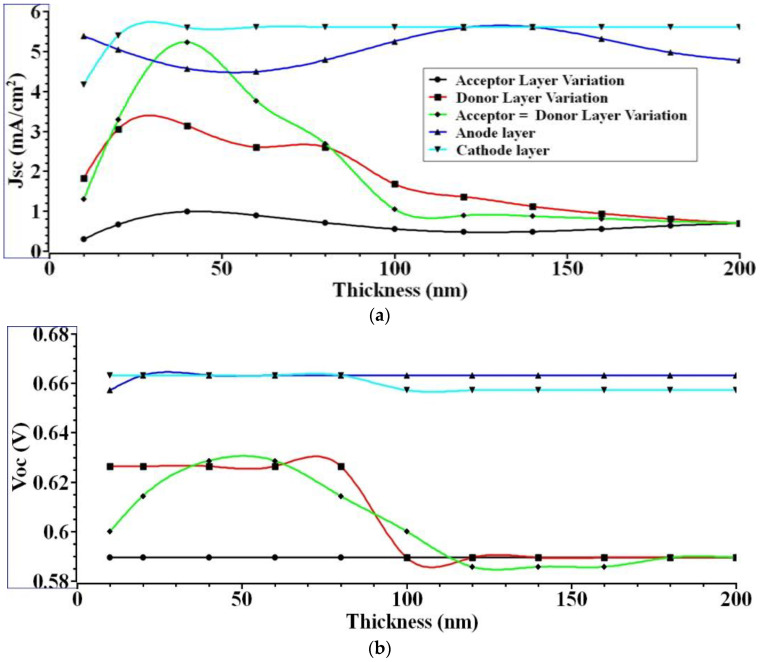

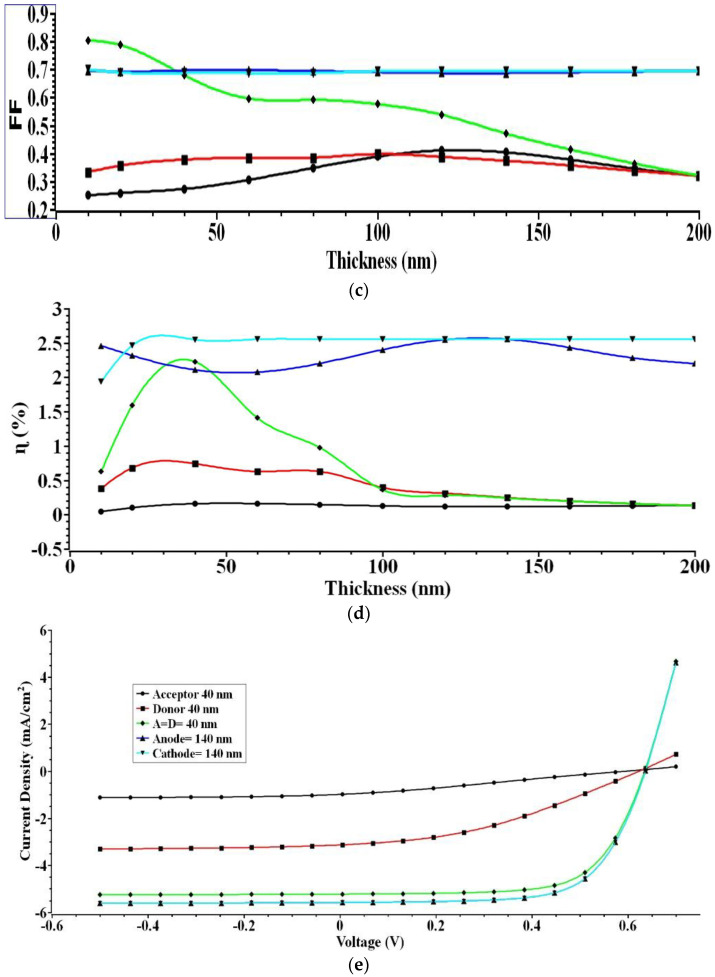
(**a**) *J_sc_* (**b**) *V_oc_* (**c**) *FF* (**d**) *ɳ* (**e**) JV curve. Illustrates the output extracted for various layer thicknesses.

(i) Initially, the thickness of the acceptor layer varied between 10 nm and 200 nm, the remaining layers, such as donor 200 nm, anode 100 nm, and the cathode layer 100 nm, remained constant.
(ii) Likewise, in the following case, the thickness of the donor layer varies from 10 nm to 200 nm, the remaining layers, such as acceptor 200 nm, anode 100 nm, and the cathode layer 100 nm, remained constant.
(iii) Finally, the thickness of the donor and acceptor layers, which together make up the active layers, is evenly altered over the range of 10 nm to 200 nm, the remaining layers, such as the anode at 100 nm and the cathode layer at 100 nm, remained constant. According to the simulation findings, the optimal active layer (Donor = Acceptor) thickness is 40 nm, with the resulting values being *J_sc_* = 5.22 mA/cm^2^, *V_oc_* = 0.63 V, *FF* = 68%, and *ɳ* = 2.3%. Figure 3a demonstrates a donor–acceptor active layer thickness over 40 nm is useless.

This number is proportional to the ratio of excitons formed during charge dissociation to those that are recombined. Since the recombination rate is inversely proportional to *J_sc_*, the drop in *J_sc_* indicates that most excitons generated in the active layer are recombining prior to hitting the p-n junction. Therefore, when the thickness of the active layer is raised, the current density drops. Figure 3b shows that for PHJ, the value of *V_oc_* falls somewhat with changes in the thickness of the active layer. The *V_oc_* value is based only on the energy gap between the donor HOMO and the acceptor LUMO. As demonstrated in Figure 3c, the FF for PHJ will drop significantly as the active layer thickness increases above 40 nm. The maximum power from an I–V curve is proportional to the FF. As the active layer becomes thicker, however, it starts to drop. Figure 3d depicts the relationship between active layer thickness and efficiency. An OSC’s efficiency is measured as the ratio of its output power to its input power. The *FF*, *V_oc_*, and *J_sc_* all have a role in the output power. Device efficiency decreases with decreasing values of *V_oc_*, *J_sc_*, and *FF*. When the thickness of the donor and acceptor active layers is increased above 40 nm, the efficiency drops. The data show that *V_oc_*, *J_sc_*, and *FF* are all crucial to achieving optimal efficiency. When the active layer’s thickness increases, carrier recombination phenomena become more prominent, reducing the carriers’ lifetime. Additionally, space charge becomes increasingly critical as device thickness increases, resulting in field-free zones with diminished collection efficiency.

The authors then realized a second scenario, in which the anode layer thickness has been changed from 10 nm to 200 nm. In comparison, the donor and acceptor layers remained at 40 nm, and the cathode layer was maintained at 100 nm, depending on the previous instance’s findings. The results show that the optimal anode layer thickness is 140 nm when considering *J_sc_* = 5.6 mA/cm^2^, *V_oc_* = 0.66 V, *FF* = 69%, and *ɳ* = 2.55%. Figure 3a displays the simulation results showing that as anode layer thickness is increased up to 140 nm, *J_sc_* rises and then falls above that thickness. High light transmittance and low sheet resistance for the model generate a rise in the *J_sc_* when the anode thickness is increased to 140 nm. As the anode thickness is increased from 140 nm to 200 nm, the *J_sc_* decreases because of the reduced light transmission. The relationship between *V_oc_* and PHJ anode layer thickness is seen in Figure 3b. Figure 3b demonstrates that the *V_oc_* value is independent of the anode layer thickness. Thus, the thickness of the anode layer has no role in the exciton formation. The *V_oc_* may be computed from the energy bandgap differences between the materials in the active layer and the anode using Equation (1), and it is true that *V_oc_* is independent of anode layer thickness. The effect of *FF* on PHJ anode layer thickness is seen in Figure 3c. It demonstrates that *FF* is independent of the anode layer thickness. Increases in efficiency are shown when the anode thickness rises from 10 nm to 200 nm, due to the increased light transmission that results. Low sheet resistance is achieved by increasing the anode thickness. When the anode thickness is increased from 140 nm to 200 nm, efficiency drops because of the reduced light transmission. In Figure 3d, the device’s efficiency drops along with the other variables as they are decreased.

Based on the findings of the first two instances, this work further extended for the third scenario in which the thickness of the cathode layer varied from 10 nm to 200 nm, while the thicknesses of the donor and acceptor layers were held constant at 40 nm and 140 nm, respectively. The simulation results show that the best performance is attained with a cathode layer thickness of 140 nm, as measured by *J_sc_* = 5.6 mA/cm^2^, *V_oc_* = 0.66 V, *FF* = 69%, and *ɳ* = 2.55%. Figure 3e depicts the JV curve for the best performance produced by each layer thickness variation. The cathode is located at the solar cell’s bottom. That is why it has a negligible impact on optical absorption. The cathode is responsible for charge collection.

### 4.2. Analyzing the PHJ Performance in Relation to the Interface Layer

The donor–acceptor interface layer in PHJ device of OSC is vital, this layer contributes significantly to the device’s overall efficiency. There is a strong correlation between the efficacy of a photovoltaic device and the optimization of donor–acceptor interface layers.

**Figure 4:**
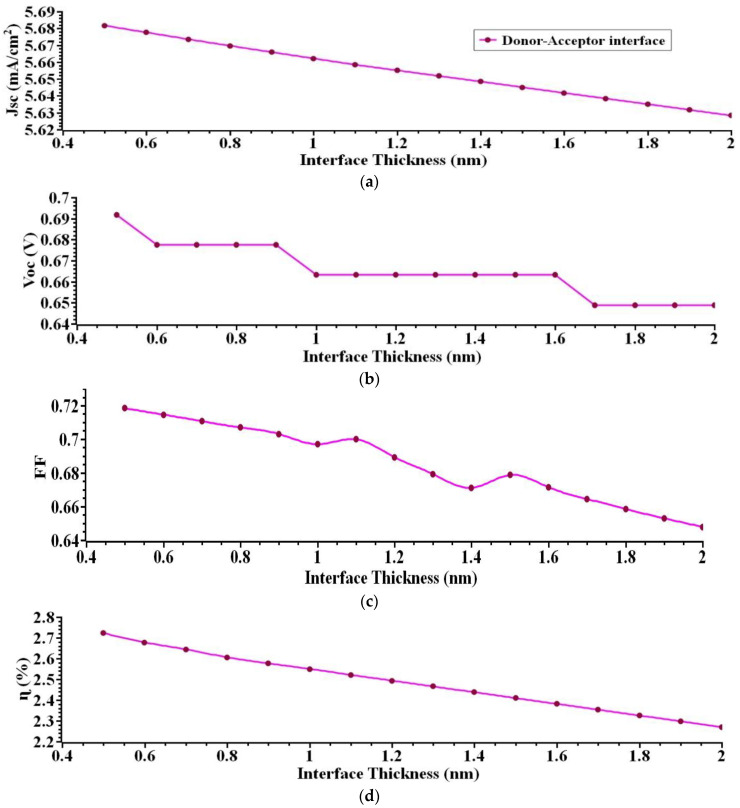
(**a**) *J_sc_* (**b**) *V_oc_* (**c**) *FF* (**d**) *ɳ*. Illustrates the output extracted for various interface layer thicknesses.

The donor–acceptor interface layer is critical, especially for PHJ devices as it affects the overall performance of the device. Excitons are dissociated at the donor–acceptor interface. Recombination of charge carriers also takes place here. Recombination current is directly proportional to interface thickness. The donor–acceptor interface is diffuse and has a finite thickness. In this research work, the effects of the donor–acceptor interface on each photoelectric conversion process are analyzed by varying the donor–acceptor interface thickness in PHJ systems. Initially, the donor–acceptor interface thickness layer is varied from 0.5 nm to 2 nm, the remaining layers’ such as acceptor 40 nm, donor 40 nm, anode 140 nm, and the cathode layer 100 nm kept constant for this analysis. Figure 4 illustrates the comparison plot characteristics of PHJ device for various donor–acceptor interface layer thicknesses from the J-V curves. As per the simulation findings, the best performance is obtained for the donor–acceptor interface layer thickness of 0.5 nm *J_sc_* = 5.6 mA/cm^2^, *V_oc_* = 0.66 V, *FF* = 69%, and *ɳ* = 2.55%. Figure 4 depicts the effect of *J_sc_, V_oc_, FF*, and interface layer thickness. As shown in Figure 4, increasing the interface layer thickness obviously significantly influences *J_sc_, V_oc_*, *ɳ*, and *FF*. Increasing the interface layer thickness reduces the device’s overall performance.

### 4.3. Effectiveness of PHJ in Optimizing Active Layer Charge Carrier Mobility

The mobility charge carriers in the PHJ device’s OSC are useful to determine the device’s functionality. Therefore, building a photovoltaic device in which the mobility of charge carriers is optimized is essential.

**Figure 5:**
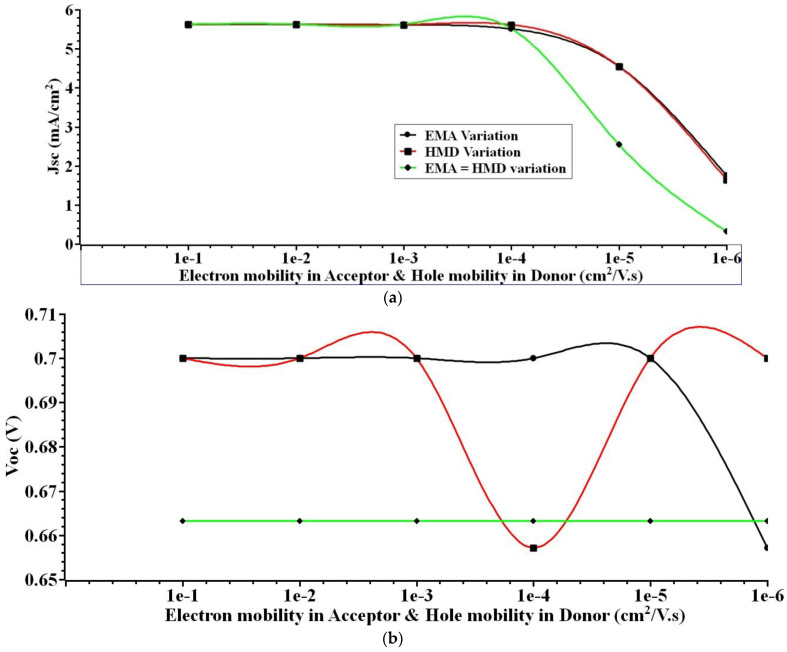

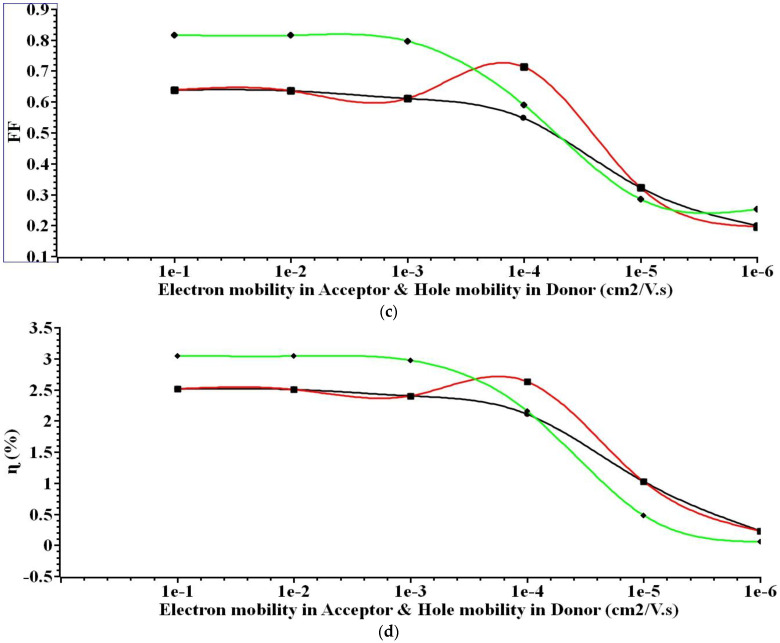
(**a**) *J_sc_* (**b**) *V_oc_* (**c**) *FF* (**d**) *ɳ*. Illustrates the output extracted for various mobility charge carriers.

The PHJ OSC’s active layer is bi-layered, including donor and acceptor layers. The work function and conductivity at the active layer of an OSC are tunable by simply adjusting the doping concentration in that layer [49]. That is why it is simple and effective to manipulate the mobility of charge carriers triggered at the heterojunction of the donor–acceptor interface layer. By controlling the mobility of electrons and holes in the active layer, researchers may tailor the s-SWCNT/C_70_ PHJ OSC performance characteristics. After being generated by light, excitons diffuse to the heterojunction interface in the active organic layer of the cell, where they dissociate into free charge carriers (electrons and holes). The extraction and recombination of charges in an OSC cell are related to the mobility of charge carriers. An increase aids carrier extraction in carrier mobility, which boosts carrier transport and strengthens bimolecular recombination. Charge carriers with low mobility tend to accumulate in a cell, whereas those with high mobilities are more easily extracted, leading to a lower charge carrier density as a result of faster carrier extraction and, hence, a lower *V_oc_* for the solar cell. Additionally, the risk of carrier recombination rises with the accelerated extraction of charge carriers. Therefore, the value of external quantum efficiency (EQE) in organic solar cells does not see much improvement in the event of a substantial increase in carrier mobility; instead, a modest increase in EQE happens in such a circumstance. Here, researchers explored the impact of varying the electron and hole mobility on the performance of an s-SWCNT/C_70_ planar heterojunction solar cell. Changes in electron and hole mobility were used to simulate the effects of these charge carriers on an OSC, the results of which are displayed in Figure 5.

**Figure 6:**
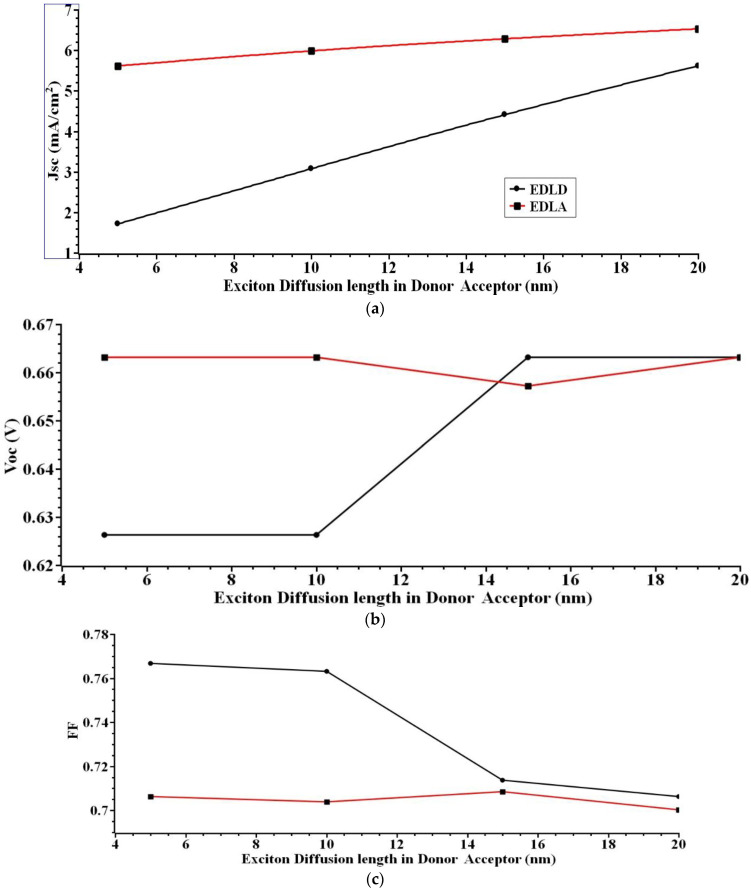

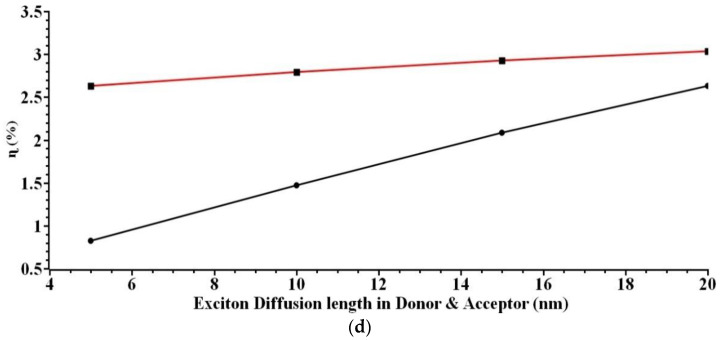
(**a**) *J_sc_* (**b**) *V_oc_* (**c**) *FF* (**d**) *ɳ*. Illustrates the output extracted for various exciton diffusion lengths.

It can be concluded that when electron mobility and hole mobility in the active area of the OSC increases, the cell’s *J_sc_*, *V_oc_*, *ɳ*, and *FF* rise. However, it is also noted that the gain in efficiency and other performance characteristics is minor for large levels of carrier mobility. The performance characteristics of the cell are affected by increasing charge carrier recombination and reducing *V_oc_* for high mobility. Because of this, improvements in solar cells *J_sc_*, *V_oc_*, *ɳ*, and *FF* are limited. Figure 6 shows the performance parameters of the s-SWCNT/C_70_ solar cell for varying electron and hole carrier mobility. The simulation results show that *J_sc_* = 5.6255 mA/cm^2^, *V_oc_* = 0.66 V, *FF* = 82%, and *ɳ* = 3.045%, the best performance is achieved for the variation in mobility charge carriers in the active layer, with electron mobility in the acceptor and hole mobility in the donor both at 1e^−1^ (cm^2^/V. s).

The effect of varying electron and hole mobility on the solar cell’s performance parameters has been analyzed. Since an electron and a hole have the same effective mass, the chance of carrier recombination grows as the mobility of electrons and holes increases. As the mobility of electrons and holes increases, the organic solar cell’s *J_sc_*, *V_oc_*, *ɳ*, and *FF* all increase.

### 4.4. Effect of Exciton Diffusion Length on the Efficiency of the s-SWCNT/C_70_ PHJ

The performance of the PHJ device OSC is highly dependent on the exciton diffusion length. In order to create a highly effective photovoltaic device, it is crucial to optimize the amount of time that excitons may diffuse through the material. By employing spectrally resolved photoluminescence quenching (SRPLQ) methods [50], it can determine the exciton diffusion length in s-SWCNT/C_70_ in relation to the crystalline order. Because crystallinity increases the exciton diffusion length in the organic semiconductor, it is useful to know whether an organic solar cell’s donor or acceptor layer is increasing the crystallinity. In this work, the performance and efficiency of the organic solar cell have been realized by increasing the exciton diffusion length.

Figure 6 shows the variation of exciton diffusion length of both donor and acceptor material, ranging from 5 to 20 nm. For this research work analysis, the donor material is kept constant as 20 nm and the acceptor material are varied from 5 to 20 nm. Then, the acceptor material is kept constant as 20 nm and donor material are varied from 5 to 20 nm. The simulation results show that *J_sc_* = 6.5295 mA/cm^2^, *V_oc_* = 0.66 V, *FF* = 70%, and *ɳ* = 3.032%, the best performance is achieved for the variation in exciton diffusion length, with exciton diffusion length in the acceptor and exciton diffusion length in the donor both at 20 nm.

A larger value of the diffusion length of photo-generated excitons has been determined to yield the best performance in PHJ OSC. With an increased exciton diffusion length, more charge carriers (electrons/holes) are generated and collected at the ITO anode/Al cathode terminal before the photo-generated exciton is recombined. This results in a larger output power per/unit area.

### 4.5. Analysis of s-SWCNT/C_70_ PHJ for Varying the Bio-Molecular Recombination Coefficient

The bi-molecular recombination coefficient strongly influences the performance of the organic photovoltaic cell used in PHJ devices. As a result, optimizing the bi-molecular recombination coefficient is crucial to producing a high-performance solar system. Only at the donor–acceptor interface does recombination of charged carriers take place. The bi-molecular recombination coefficient characterizes the intensity of recombination at the interface.

**Figure 7:**
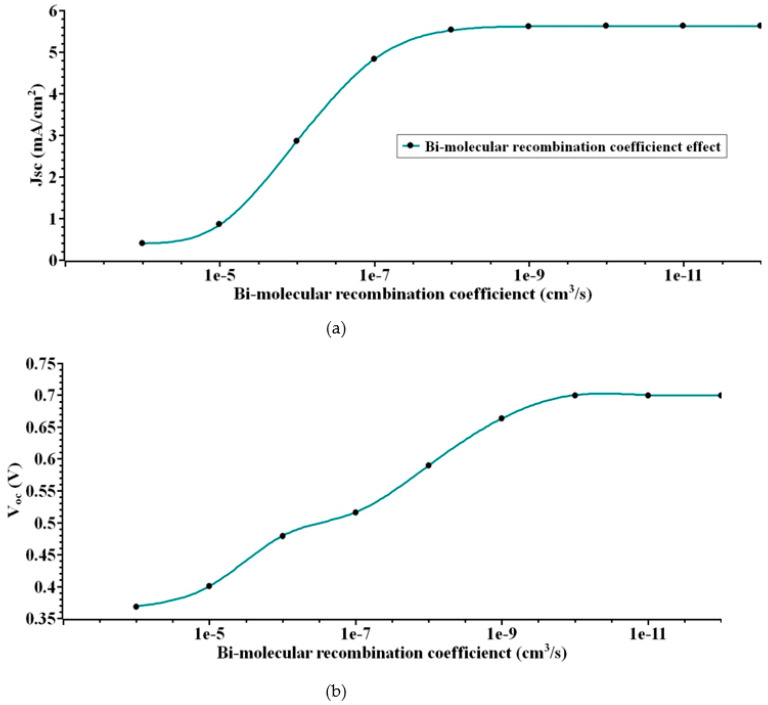

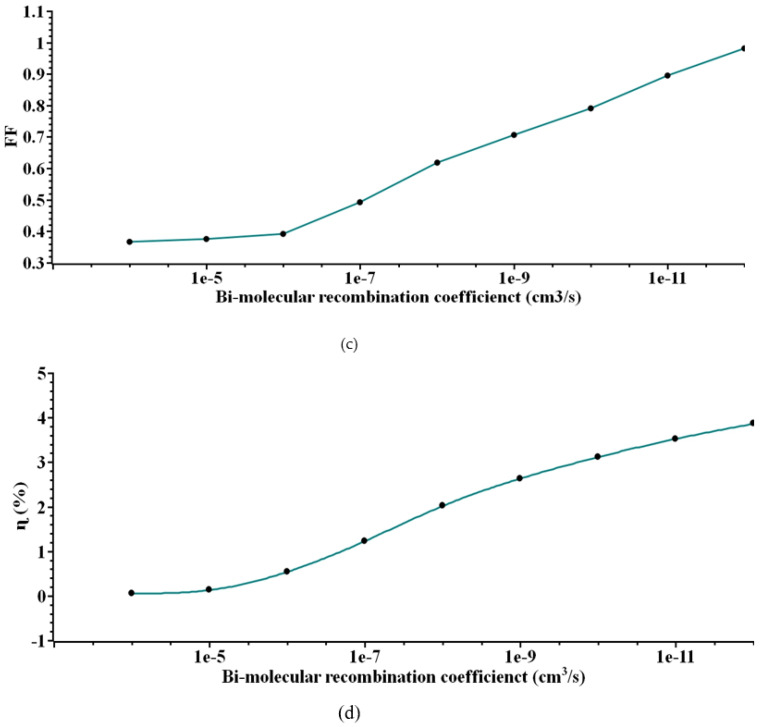
(**a**) *J_sc_* (**b**) *V_oc_* (**c**) *FF* (**d**) *ɳ*. Illustrates the output extracted for various bi-molecular recombination coefficients.

Koster at. al. later demonstrated how increasing the Bi-molecular recombination coefficient in PHJ systems affects the individual photoelectric conversion processes [51,52,53]. To begin, the researcher introduces a range of values for the bi-molecular recombination coefficient, from 1 × 10^−4^ nm to 1 × 10^−12^ nm. Figure 7 illustrates the comparison plot characteristics of PHJ device for various bi-molecular recombination coefficients from the J-V curves [54]. From the results it has been observed that a highest performance is achieved for the bi-molecular recombination coefficient of 1 × 10^−12^ *J_sc_* = 5.6 mA/cm^2^, *V_oc_* = 0.7 V, *FF =* 98%, and *ɳ* = 3.9% is obtained. However, as the actual bi-molecular recombination coefficient of the active layer material is 1 × 10^−10^, the obtained output is *J_sc_* = 5.61 mA/cm^2^, V_oc_ = 0.7 V, *FF* = 79%, and *ɳ* = 3.1%.

### 4.6. Comparison Analysis of PHJ Devices

Table 1 compares the proposed PHJ structure results with the previously reported works. From this table it clearly indicates that the proposed active layer device shows much better and equivalent performance compared to the previously reported works.

## 5. Conclusions and Future Recommendation

This analysis suggests a practical path toward the efficient deployment of OPV by suggesting ways to optimize their attributes, which are extremely dependent on OSC’s performance. The collected information allows for the assessment that performance has been enhanced. In conclusion, it was determined that its performance was superior.

Here, the electrical and optical properties of the ITO/s-SWCNT/C_70_/Al PHJ OSC will be analyzed to determine its performance parameters. The ITO/s-SWCNT/C_70_/Al cell’s current–voltage characteristics were investigated by altering the layer thickness, exciton diffusion length, bi-molecular recombination coefficient, and carrier mobility, respectively. In each instance, the *J_sc_*, *V_oc_*, *ɳ*, and *FF* of the cell have been calculated and examined. The simulation has been run using 1 kW/m^2^ of incident solar irradiance, 1.5 A.M of air density, and with ITO and Al serving as the anode and cathode of the s-SWCNT/C_70_ solar cell, respectively. The *J_sc_*, *V_oc_*, *ɳ*, and *FF* of the solar cell were calculated by varying the layer thicknesses from 10 nm to 200 nm, and from 0.5 nm to 2 nm for interface layer thickness, as well as varying the exciton diffusion length, electron mobility, and hole mobility, and also the bi-molecular recombination coefficient of the material.

The findings show that if the device is fabricated with an optimized structure, high exciton diffusion length, and increased carrier mobility, especially hole mobility at the donor layer of the cell, a highly efficient ITO/s-SWCNT/C_70_/Al PHJ solar cell may be generated. It was determined from the data that the electrical power output is increased because the thin donor and acceptor layers allow for more absorption of charge carriers at the active layer and suffer less from the recombination effect. Therefore, a thin donor layer with enhanced exciton diffusion length should be produced in order to create a highly efficient ITO/s-SWCNT/C_70_/Al PHJ solar cell with a high output power density. Results also imply that charge carrier mobility must be increased by modifying the doping concentration of the donor–acceptor layer of the cell to achieve high rates of charge carrier diffusion in the active layer. Increased charge carrier mobility also results in minimal carrier losses in the active layer and high electrical power generation. For exciton diffusion lengths of 20 nm for donor and 5 nm for acceptor, donor thicknesses of 40 nm, acceptor thicknesses of 40 nm, anode thicknesses of 140 nm, cathode thicknesses of 100 nm, interface layer thickness of 0.5 nm, the electron mobility in the acceptor and hole mobility in the donor both at 1e^−1^ (cm^2^/V. s), with bi-molecular recombination coefficient of 1 × 10^−12^ achieved the maximum performance in this work.

In the future, the features of this device will be evaluated with the findings, following which the device will be improved for fabrication employing these nanomaterials. The device will also have been implemented for potential usage in micro-devices for future use in biomedical and commercial applications.

## Figures and Tables

**Table 1:** Comparison of PHJ experimental work with the present work.

Ref.	Active Layer Material (Donor–Acceptor) Utilized	PCE
[43]	P3HT/PC_61_BM	3.5%
[55]	Tetra Benzo-Porhyrin (BP)/PC_61_BM	2.2%
[56]	PSDTTT/PC_61_BM	3.8%
[57]	PCDTBT/PC71BM	2.11%
[58]	PTB7/N2200	2.94%
This work	s-SWCNT/C_70_	3.9%

## Data Availability

All data and material used to prepare this manuscript are available in this document.
